# An Efficient Lightweight Method for Steel Surface Defect Detection

**DOI:** 10.3390/s25247527

**Published:** 2025-12-11

**Authors:** Aiyun Zheng, Xinyu Jiang, Weimin Liu

**Affiliations:** College of Mechanical Engineering, North China University of Science and Technology, Tangshan 063210, China

**Keywords:** defect detection, LDConv, lightweight, loss function

## Abstract

Surface defects are inevitable in the production of steel. However, traditional methods in industrial production face great challenges in detecting complex defects. Therefore, we propose LCED-YOLO based on YOLOv11 for steel defect detection. Firstly, an edge information enhancement module, C3K2-MSE, is designed to strengthen the extraction of edge information. Secondly, LDConv is introduced to lightweight the neck structure and reduce parameters. Then, a lightweight decoupling head designed for model detection tasks is proposed, further achieving model lightweighting. Finally, by introducing a learnable attention factor to optimize the CIoU loss, we focused on locating difficult samples, enhancing the detection capability. A large number of experiments were conducted on the NEU-DET and GC10-DET datasets. Compared to YOLOv11, the mAP50 of the proposed model improved by 2.6% and 3.3%, attaining 79.8% and 70.3%, respectively. It decreased 19% of parameters and 23% of floating-point operations, fulfilling the needs of lightweight and detection precision.

## 1. Introduction

Deformation damage is inevitable in the actual production and manufacturing of steel, which causes defects such as crazing, pitted surface, and inclusions [1]. These defects significantly impact the quality of steel products, causing safety concerns and economic losses for companies. In the process of steel production, it is necessary to find and eliminate product defects in time, so as to improve the safety of products and avoid economic losses [2]. The traditional method is mainly the visual detection method [3], which is a manual detection method. Manual visual inspection methods have the disadvantages of low detection efficiency and limited accuracy. The limitations of the inspectors themselves can also lead to product misdetection and missed detection.

With the advancements in deep learning and computer vision, deep learning-based defect detection methods are increasingly being applied to various detection tasks. Deep learning methods have great advantages in detection speed and accuracy. Researchers have started employing these methods to solve different defect detection tasks in production.

Currently, deep learning-based detection methods are divided into one-stage methods and two-stage methods. Among one-stage methods, Deng et al. [4] introduced the LFD-YOLO model, which is designed to detect surface defects on engine turbine blades. To detect printed circuit board (PCB) defects, Jiang et al. [5] introduced dilated convolution and coordinate attention into the Single-Shot MultiBox Detector (SSD). Li et al. [6] embedded an Efficient Channel Attention (ECA) mechanism into the YOLOX model to realize wood surface defect detection. Xing et al. [7] constructed different scale convolution layers in the backbone network to detect the defects of railway train wheels. Xiang et al. [8] used the HookNet model to detect fabric defects. Chen et al. [9] designed a lightweight network, YOLOv8-FSD, for detecting surface defects on photovoltaic cells. Gao et al. [10] introduced LGR-Net, a method for detecting defects in elevator guide rail clamps. This method employs a small object detection layer to build a multi-scale feature fusion network, thereby attaining greater detection accuracy. In two-stage methods, Chen et al. [11] proposed an MANet network model applied to road defects detection based on MobileNet. Xiao et al. [12] proposed a model for tiny target detection. In the model, the context enhancement module (CEM) is responsible for strengthening target feature information, and the feature purification module (FPM) is responsible for eliminating conflicting feature information. Tan et al. [13] introduced an image processing method that they integrated into their proposed segmentation algorithm, thereby enabling the automatic identification of tunnel water leakage. Cui et al. [14] proposed a CAB-Net, which uses dilated convolution to enhance target context information and improves ability to detect tiny targets. Urbonas et al. [15] used Faster R-CNN to identify defects on the surface of wood panels.

Although deep learning techniques offer advantages in various defect detection tasks, they still face significant challenges and limitations in practical industrial applications. Firstly, the complexity of surface defects brings some challenges to detection. Secondly, the accuracy, parameter quantity and inference speed of defect detection models are also very important in practical applications [16].

In view of the issues present in existing detection methods, we proposed a detection model named LCED-YOLO. The model aims to enhance model performance while maintaining a lightweight structure. First of all, we designed a module to enhance the model’s extraction capability. Secondly, LDConv was incorporated into the network bottleneck, which decreases the model’s parameters. Thirdly, we developed a lightweight decoupled head specifically for detection tasks, further lowering the model’s parameters. Finally, by introducing a learnable factor to optimize the CIoU loss function, the sample imbalance issue is addressed, and detection performance is strengthened. The main contributions are as follows:

1. A multi-scale enhancement module (MSE module) was designed in conjunction with the C3K2 module to create the C3K2-MSE module, which effectively enhances the feature information processing capability by accentuating edge information within the features.

2. A lightweight neck network and detection head were designed. In comparison to the original network, this approach reduces both the parameter count and FLOPs, while simultaneously enhancing detection accuracy.

3. By employing Focal-CIoU as the model’s loss function, the introduction of learnable factors effectively mitigates the sample imbalance problem in detection tasks, thereby enhancing the model’s detection performance.

The rest of this article is organized as follows. In Section 2, the related research work is introduced. Section 3 introduces the proposed LCED-YOLO network model in detail. Section 4 presents the experimental evaluation. Finally, Section 5 summarizes the work presented in this paper.

## 2. Related Works

### 2.1. Object Detection

With the development of target detection, a large number of excellent target detection algorithms have emerged. Most of them are high-efficiency, high-precision, and general-purpose target detection frameworks, which can solve most practical target detection problems. In general, deep learning-based object detection methods are categorized into two main frameworks: one-stage methods and two-stage methods.

The one-stage method directly obtains the target’s class and location coordinates, representing algorithms include SSD [17] and You Only Look Once (YOLO) models [18,19,20]. The two-stage method generates candidate boxes and then classifies the candidate box areas, representing algorithms include Region-based Convolutional Neural Network (RCNN) [21], fast RCNN [22], and mask RCNN [23]. Because the two-stage algorithm first generates the candidate boxes and then reclassifies them, the first-stage algorithm has more advantages in detection speed.

In the two-stage detection method, Ma et al. [24] designed a method to improve robotic picking accuracy based on the Mask RCNN object detection algorithm. Han et al. [25] employed an enhanced Faster-RCNN model to detect traffic signs. Qi et al. [26] used an enhanced Fast-RCNN for accurate target detection. In the one-stage detection method, Chen et al. [27] employed an enhanced SSD model to accurately detect small vehicles; Liu et al. [28] proposed the lightweight network YOLO-BFRV, designed to enhance the detection of PCB defect by expanding the receptive field. Chen et al. [29] used the improved YOLOv3 to complete face detection. By changing the regression loss function, the model detection results are more accurate. Yi et al. [30] applied the YOLOv5 model to detect pedestrian helmets.

### 2.2. Defect Detection

As deep learning technology continues to develop and advance, a growing number of researchers are applying it to defect detection tasks. The manual feature extraction of traditional methods relies heavily on subjective experience, resulting in a low recognition rate for various defects. The deep learning method extracts strong representative features through neural network, locates and classifies defects, and achieves excellent results in defect detection.

Many defect detection studies are based on classic object detection frameworks, such as the YOLO series and Fast R-CNN. For example, Xie et al. [31] detected steel surface defects based on Fast R-CNN. Yu et al. [32] proposed an efficient detection network based on YOLO. Zhao et al. [33] proposed a detection model RDD-YOLO. Liu et al. [34] realized the detection of surface defects on aero-engine blades by improving Faster R-CNN. Chen et al. [35] designed a lightweight CenterNet model that enhances defect features by leveraging contextual information, resulting in improved detection performance. Zhao et al. [36] obtained a lightweight network model by fusing ShuffleNet and SSD models to detect turbine blade defects.

Two-stage detection methods offer high accuracy, but their large model size and slower detection speed make them less suitable for industrial production. One-stage methods directly acquire target category and location information, enabling faster detection. Therefore, this paper proposes a one-stage algorithm specifically designed for steel surface defect detection.

## 3. Methods

### 3.1. YOLOv11 Network Architecture

Due to variations in network depth, width, and model parameters, YOLOv11 is currently available in five versions: YOLOv11n, YOLOv11s, YOLOv11m, YOLOv11l, and YOLOv11x. The C3K2 module is a residual module, which integrates C2f and C3 modules, and applies the C3K structure internally based on the C2f module structure. The SPPF module processes features by substituting a pooling layer with a single large pooling kernel with pooling layers that use multiple smaller pooling kernels, thereby reducing the computational load while maintaining the capability to integrate multi-scale features. The C2PSA module is an extension of the C2f module, which combines the Pointwise Spatial Attention (PSA) mechanism [37], retaining the global information of the features. The neck uses the PAN-FPN structure [38] for feature information fusion. As the prediction part of the model, the head structure can effectively present the target position and category. YOLOv11 employs depthwise separable convolution (DWConv) [39] within its head branch for feature fusion, thereby reducing redundant calculations. This paper’s model is enhanced from YOLOv11n to increase the precision of steel surface defect detection and to lower the challenges associated with model deployment.

### 3.2. The Proposed LCED-YOLO

**Figure 1:**
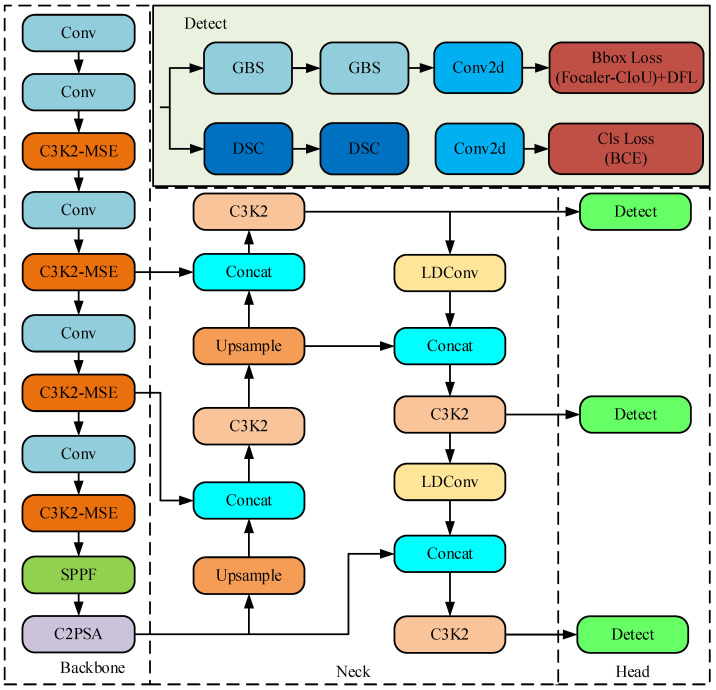
LCED-YOLO network architecture.

The proposed LCED-YOLO is introduced. LCED-YOLO belongs to the one-stage detection network, as shown in Figure 1.

In the backbone network, an edge extraction module was designed to accurately extract information about edge defects on the steel surface. By constructing the correlation between local and global feature information, the information expression is effectively enhanced. For the neck network, we introduced the LDConv lightweight module to reduce the model’s parameters and computational complexity. In the head part, the task is decoupled so that the learned features are orientated to the corresponding tasks. Next, the proposed LCED-YOLO network model structure will be introduced in detail.

#### 3.2.1. Backbone

**Figure 2:**
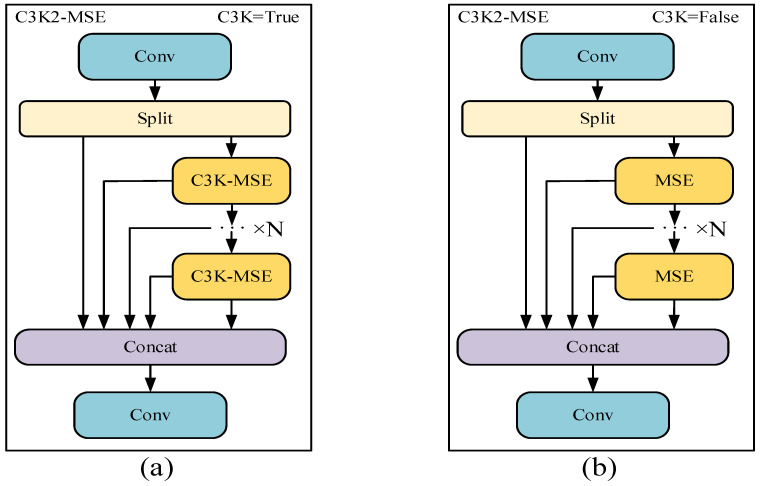
C3K2-MSE module structure. (**a**) The C3K-MSE module is utilized when C3K is set to True; (**b**) the MSE module is utilized when C3K is set to False.

Steel surface defects vary greatly in position and shape, while CNNs have limitations in effectively modeling long-range dependencies, which restricts the ability to capture features. Therefore, in the face of changeable defect detection tasks, the model often fails to achieve the expected results. To improve the ability to extract complex defects in steel, the C3K2-MSE module is designed by introducing a multi-scale edge information enhancement module into C3K2, as shown in Figure 2.

**Figure 3:**
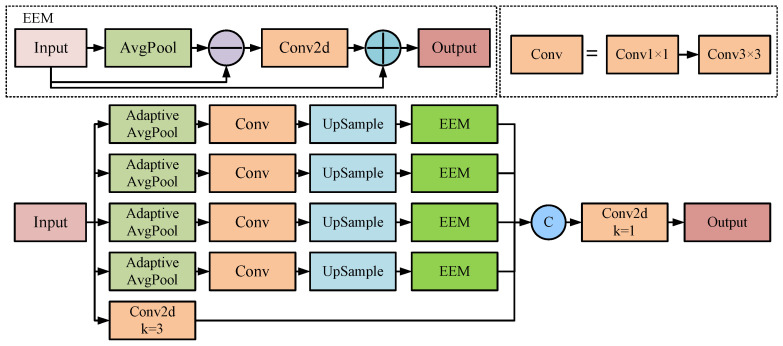
MSE module structure.

Inspired by the idea of fusing different scale features from the RFB structure [40], this paper proposes a multi-scale edge information enhancement module. First, multi-scale pooling is applied to the input features by performing adaptive average pooling with kernel sizes of 3, 6, 9, and 12, respectively, to extract features at various scales. This approach effectively captures the image’s multi-level feature information. Secondly, the feature map is adjusted by successively applying two convolutions with 1 × 1 and 3 × 3 kernel sizes. Subsequently, an upsample operation is employed to align features from different scales to the same spatial resolution. Thirdly, the edge enhancement module (EEM) is responsible for enhancing the model’s sensitivity to edge features. Subsequently, the processed multi-scale features and local features are spliced, and the convolution is fused into a unified feature, as shown in Figure 3.

The EEM retains the effective feature information while enhancing the edge information. By strengthening the edge information, the defect edge is clearer, and the edge feature enhancement effect is achieved. Firstly, the pooling operation is performed on the input features to remove noise and detail information in the image and retain its low-frequency information. Secondly, the difference between original and smoothed features is calculated to obtain the edge feature information. These extracted features are further processed by the convolution operation, thereby highlighting defect edge details. Finally, the edge-enhanced features are obtained by integrating the refined edge details with the original information. This process can be formalized as shown in Equations (1) and (2).

(1) $$F_{i}=X-AvgPool(X),$$

(2) $$F_{out}=X+Conv_{1\times 1}(F_{i}),$$

where X denotes the input feature, $F_{i}$ denotes the edge feature, and $F_{out}$ denotes the edge-enhanced feature.

In summary, the MSE module can effectively integrate the extracted multi-scale edge information, improve the flexibility of the model, and provide an efficient solution.

#### 3.2.2. Neck

In the model neck network, standard convolution is used for down-sampling. Standard convolution uses fixed sampling positions for feature extraction, which is limited to local windows and cannot effectively capture global information. To overcome this, we introduce Linear Deformable Convolution [41] (LDConv), which dynamically adjusts the position of sampling points through the characteristics of LDConv, so that it can adapt to the geometric shape and texture changes in input features and improve the precision of feature extraction.

**Figure 4:**
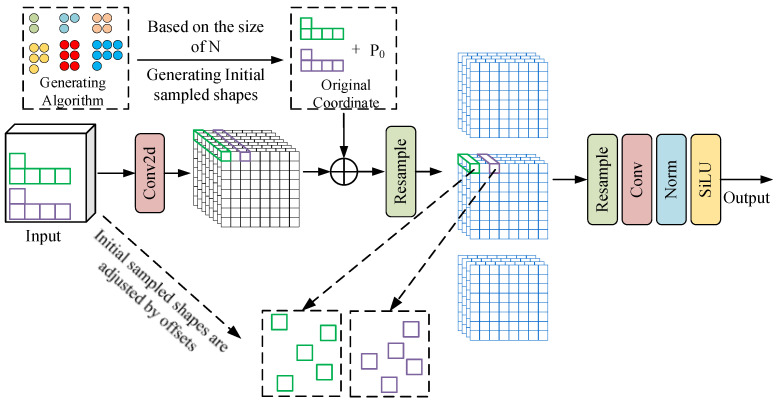
LDconv structure diagram.

LDConv is shown in Figure 4. Firstly, according to the convolution kernel size, the initial sampling coordinate is generated. Regular or irregular coordinates are then produced by calculating the quantity of convolution kernels. The corresponding convolution operation at the position is shown in Equation (3).

(3) $$Conv(P_{0})=\sum_{P_{i}\in R}w_{P_{i}}\times x(P_{0}+P_{i}),$$

where R, w and $x(P_{0}+P_{i})$ denote the generated sampling grid, convolution parameters, and the pixel values at their respective spatial locations.

Secondly, the offset of the corresponding convolution kernel is obtained according to the convolution operation, and these offsets dynamically adjust the sampling positions of the kernel. Specifically, these offsets are learned from the input features through convolution operations, and the offsets are added to the original coordinates to generate new sampling coordinates to achieve accurate modeling of the geometric features of the target. Finally, according to the adjusted sampling position, the features are resampled, and the resampled feature map is shaped. After convolution, normalization, and the SiLU activation function, a new output feature map is obtained.

LDConv can dynamically generate the sampling shape according to the change in the target shape, enabling convolutional kernels to adapt based on feature content and effectively enhance the ability to capture irregular targets. Compared with the traditional convolution kernel fixed-sampling-point method, LDConv makes the sampling point distribution more flexible by learning the offset, so as to better deal with targets with complex boundaries or irregular shapes. In addition, LDConv avoids the computational burden of quadratic growth of parameters in traditional deformable convolution by designing parameters with linear growth, so that it has better computational efficiency while maintaining high flexibility. Therefore, the model’s PAFPN structure was redesigned by introducing LDConv (kernel size 3, stride 2) to replace the neck downsampling module, which reduced the number of model parameters.

#### 3.2.3. Head

**Figure 5:**
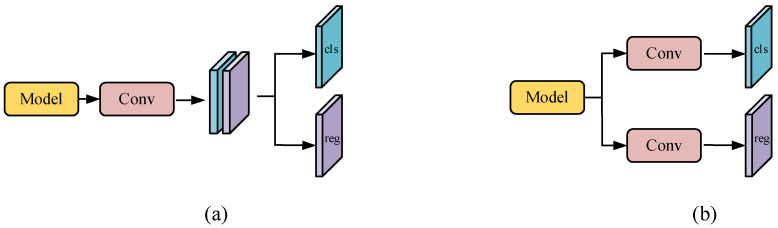
Illustration of two types of detection heads: (**a**) coupled head and (**b**) decoupled head.

In most network models, the head is usually used to complete the task of predicting target location and class, which is generally divided into the Coupled head [18,42] and Decoupled head [43], as shown in Figure 5. These two methods are used in different ways in dealing with classification and regression tasks. The coupling head uses a single network layer to complete the prediction of the target position and category, and the decoupling head completes the prediction of the target position and category through two different branches. The characteristics of the coupling head will impact classification and regression tasks, and its prediction accuracy will be affected in the face of complex defects. Therefore, we use a decoupled head for prediction.

We design a lightweight detection head. This head handles classification and regression tasks with two separate modules. For regression tasks, we propose a GBS module, and for the classification task, we use the DSC module.

**Figure 6:**
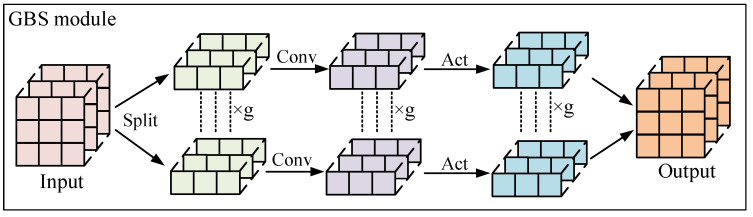
GBS model structure.

As shown in Figure 6, the GBS module initially splits the input features into g uniform segments across the channel axis. These segments are then multiplied with correspondingly partitioned convolution kernels. The processed features subsequently pass through a BN layer and activation function layer before being concatenated channel-wise to generate the new feature representation. The GBS module divides the features into groups, so that each group can be calculated independently, which effectively improves the parallelism of model calculation. Each group is only convoluted with a part of the input channel, so the model’s parameters and calculations will be reduced. The GBS module uses fewer parameters and calculations to achieve the same effect as before, which effectively reduces the overall parameter and computational complexity. The parameters and computational complexity of the original head module and GBS module are shown in Equations (4)–(7). It is evident that the GBS module reduces the parameters and computational complexity by one g and realizes the lightweight optimization of the model head.

(4) $$params=O_{H}\times O_{W}\times C_{in}\times C_{out},$$

(5) $$params=O_{H}\times O_{W}\times C_{in}\times C_{out}\times \frac{1}{g},$$

(6) $$FLOPs=2\cdot K_{H}\cdot K_{W}\cdot C_{in}\cdot C_{out}\cdot O_{H}\cdot O_{W},$$

(7) $$FLOPs=2\cdot K_{H}\cdot K_{W}\cdot C_{in}\cdot C_{out}\cdot O_{H}\cdot O_{W}\cdot \frac{1}{g}.$$

where $O_{H}$ denotes the height of the feature map, $O_{W}$ denotes the width of the feature map, $K_{H}$ denotes the height of the convolution kernel, $K_{W}$ denotes the width of the convolution kernel, $C_{in}$ denotes the number of input channels, $C_{out}$ denotes the number of output channels, and g denotes the number of groups.

**Figure 7:**
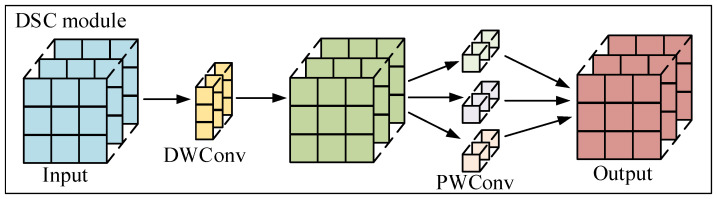
DSC model structure.

As shown in Figure 7, the DSC module is composed of DWConv and PWConv, which is responsible for processing model classification tasks. The DSC module initially employs a 3 × 3 depthwise separable convolution (DWConv) to independently process each channel of the input feature map for spatial feature extraction. Although this operation can reduce the model’s computational complexity, it fails to effectively leverage cross-channel feature correlations at identical spatial positions. Therefore, we added PWConv to deeply integrate these maps. The formalization process of the DSC module is shown in Equation (8).

(8) $$P_{i}=PWConv_{1\times 1}(DWConv_{3\times 3}(X_{i}))$$

where $X_{i}$ denotes the input feature, and $P_{i}$ denotes the output feature. In this way, the DSC module can significantly reduce the model’s parameters and computational complexity while maintaining its feature extraction ability.

#### 3.2.4. Loss Function

**Figure 8:**
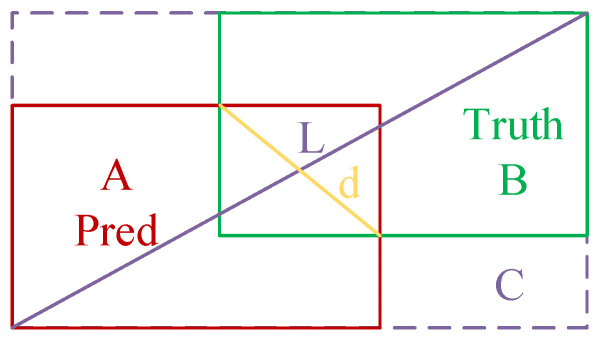
CIoU schematic.

The CIoU [44] loss function used in the original YOLOv11 model was proposed in 2020; CIoU is shown in Figure 8. By leveraging the disparity in width-to-height ratios between predicted and target boxes, the accuracy of the target regression box is enhanced. CIoU initially computes the Intersection over Union (IoU) between two objects, followed by incorporating an influence factor to account for their difference.

In the figure, C represents the minimum rectangular area containing the true box and the prediction box, A denotes the prediction box, and B denotes the true box. L denotes the diagonal length of the minimum rectangular region of C, and d denotes the Euclidean distance between the centroids of the two boxes. Therefore, the calculation formula is shown in Equations (9)–(11).

(9) $$L_{CIoU}=IoU-\frac{\rho^{2}(b,b^{gt})}{L^{2}}-\alpha \upsilon ,$$

(10) $$\alpha =\frac{\upsilon }{(1-IoU)+\upsilon },$$

(11) $$\upsilon =\frac{4}{\pi^{2}}{\left(\arctan\frac{w_{gt}}{h_{gt}}-\arctan\frac{w}{h}\right)}^{2}.$$

where $IoU=\frac{A\cap B}{A\cup B}$, b and $b^{gt}$ denote the prediction box and the true box, w and $w_{gt}$ denote their respective widths, h and $h_{gt}$ denote their corresponding heights.

While CIoU accounts for the aspect ratios of both bounding boxes, its reliance on relative difference metrics may occasionally impede the model’s ability to optimize similarity measures, particularly when handling objects with substantial scale and aspect ratio variations. In order to solve this problem, we optimize CIoU by introducing a learnable attention factor to dynamically weight different samples, thereby prioritizing those that significantly influence localization precision. The IoU was reformulated through a linear interval mapping approach, as expressed in Equation (12).

(12) $$IoU_{focaler}=\left\{\begin{array}{c} 0,\\ \frac{IoU-d}{u-d},\\ 1, \end{array}\right.\begin{array}{c} IoU<d\\ d\ll IoU\ll u\\ IoU>U \end{array}$$

where $IoU_{focaler}$ is the reconstructed Focaler-IoU, d and u∈[0,1]. The parameters d and u are adjustable. By adjusting the value of d and u, $IoU_{focaler}$ can focus on different regression samples. The definition of loss is shown in Equation (13).

(13) $$L_{focaler-IoU}=1-IoU_{focaler}$$

Therefore, the Focaler-IoU loss is applied to the CIoU loss function, and the definition of $IoU_{focaler}$ is shown in Equation (14).

(14) $$L_{focaler-CIoU}=L_{CIoU}+IoU-IoU_{focaler}$$

For the dynamic focusing of Focaler-IoU on various regression samples, it is defined as in Equation (15), where α > 0 serves as a balancing factor controlling the magnitude of the weights, and γ ≥ 0 acts as a focusing factor that dynamically adjusts the weights of easy versus hard samples. For the $\alpha \times IoU^{\gamma }$, if the focusing factor γ > 0, this term’s value increases monotonically as the IoU value increases, as demonstrated in Equation (16).

(15) $$L=-\alpha \times IoU^{\gamma }\times \log(IoU)$$

(16) $$\frac{\partial (\alpha \times IoU^{\gamma })}{\partial (IoU)}=\alpha \gamma \times IoU^{\gamma -1}>0(foranyIoU\in (0,1))$$

This indicates that the weight function can dynamically adjust the processing intensity for different samples based on the IoU. When the overlap between the predicted box and the ground truth box is low (IoU → 0+), the weight approaches zero, which enhances the gradient response of the loss function for difficult samples. Conversely, when the overlap is high (IoU → 1−), the weight stabilizes at a constant α, preventing the gradients of easy samples from dominating overall. This method makes the model focus more on difficult-to-classify bounding boxes by reducing the weights of high-confidence samples.

The incorporation of Focaler-IoU loss optimizes the model, enabling it to mitigate class imbalance challenges in detection tasks while boosting bounding box regression accuracy, ultimately elevating overall performance.

## 4. Experiments

### 4.1. Datasets

To verify the effectiveness of the proposed LCED-YOLO network, we conduct experimental verification on the NEU-DET [45] and GC10-DET [46] datasets. The NEU-DET dataset includes six distinct categories of steel surface defects, which comprise crazing (Crz), rolled-in scale (Rs), inclusions (In), patches (Pa), scratches (Sc), and a pitted surface (Ps). This study employs an 8:1:1 ratio to partition the dataset. There are 1440 images for training, 180 images for validation, and 180 images for testing. Additionally, the GC10-DET dataset includes ten categories of steel surface defects, which comprise oil spots (Os), creases (Crs), water spots (Ws), crescent gaps (Cg), silk spots (Ss), inclusions (In), weld lines (Wl), containing punching (Pu), waist folding (Wf), and rolled pits (Rp). This study employs an 8:1:1 ratio to partition the dataset, consisting of 1834 training images, 230 validation images, and 230 test images. The defects contained in these datasets are diverse and random in defect distribution and shape size. These characteristics will greatly increase the difficulty of extracting complex features of defects.

### 4.2. Experimental Setup

The experiment was completed under the Windows10 operating system, based on torch 1.12.1 and Python 3.8 as the deep learning framework implementation model. The system utilized an NVIDIA GeForce RTX 3060 GPU with 12 GB memory and an Intel Core i5-13400F CPU. The version of CUDA is 11.6. All input images are standardized to a resolution of 640 × 640, the training cycle is 300, the batch size is 8, and the SGD optimizer is used to optimize the model. The initial learning rate is 0.01, the weight attenuation coefficient is 0.0005, and the momentum is 0.937. The Mosaic method is used for data enhancement, and the last 10 epochs are closed. In order to ensure the fairness and comparability of the experiment, pre-training weights are not used in all experiments in this paper. All comparison methods and our proposed model were trained from scratch under identical training conditions.

### 4.3. Evaluation Basis

In order to evaluate the model’s performance and evaluate the effectiveness of the proposed method from different aspects, we select the average precision (AP), mean average precision (mAP), model parameters, model calculation, and FPS as the evaluation indexes of the model. The calculation formulas of these indexes are given in Equations (17)–(20).

(17) $$AP=\int_{0}^{1}P(R)dR$$

(18) $$mAP=\frac{1}{n}\sum_{i=1}^{n}AP(i)$$

(19) $$P=\frac{TP}{TP+FP}$$

(20) $$R=\frac{TP}{TP+FN}$$

where P represents the accuracy rate, which is the probability of predicting the correct number of positive samples in all predicted positive samples, and R represents the recall rate, which is the probability of predicting the correct number of positive samples in all actual samples. TP represents the number of positive samples predicted correctly, FP represents the number of positive samples predicted incorrectly, and FN represents the number of positive samples predicted as negative samples.

### 4.4. Comparison Experiments

To validate the advantages of LCED-YOLO, it was compared against several popular algorithms. The main comparison algorithms include SSD [17], YOLOv5s, YOLOv8n, YOLOv11n, Faster R-CNN [22], RT-DETR [47], and the advanced defect detection models WSS-YOLO [48] and RDD-YOLO [33]. Comparative experiments were performed on both the NEU-DET and GC10-DET datasets, which further demonstrated the efficacy and advantages of our proposed approach. In our experimental results, we employed multiple-repetition averaging to ensure the fairness of the outcomes.

#### 4.4.1. Comparisons with Other Methods on NEU-DET

According to Table 1 and Table 2, the LCED-YOLO model shows significant performance compared with other methods. Compared with YOLOv11n, LCED-YOLO has a 2.6% increase in mAP50. Although it has a slight decrease in FPS, its model achieves a 19.2% reduction in parameters and a 23.1% decrease in computational complexity. It has better comprehensive performance and can meet the lightweight requirements of industry. LCED-YOLO reaches 79.8% mAP50, which is the best for the three defects of In, Ps, and Pa compared with all the other models.

Compared with SSD, Faster R-CNN, and RT-DETR, LCED-YOLO increases mAP50 by 7.1%, mAP50 by 4.9%, and mAP50 by 5.8%, respectively. Compared with YOLOv5s and YOLOv8n, it increases mAP50 by 4.5% and mAP50 by 2.8%, respectively. Compared with RDD-YOLO, the proposed method improves mAP50 by 2.8% while paying less resources and achieves higher detection accuracy. Although mAP50 is reduced by 0.1 compared with WSS-YOLO, LCED-YOLO has greater advantages in parameter quantity and computational complexity. Compared with WSS-YOLO, the parameter quantity is reduced by 53.3% and the computational complexity is reduced by 47.9%, which is more suitable for lightweight industrial deployment tasks.

**Figure 9:**
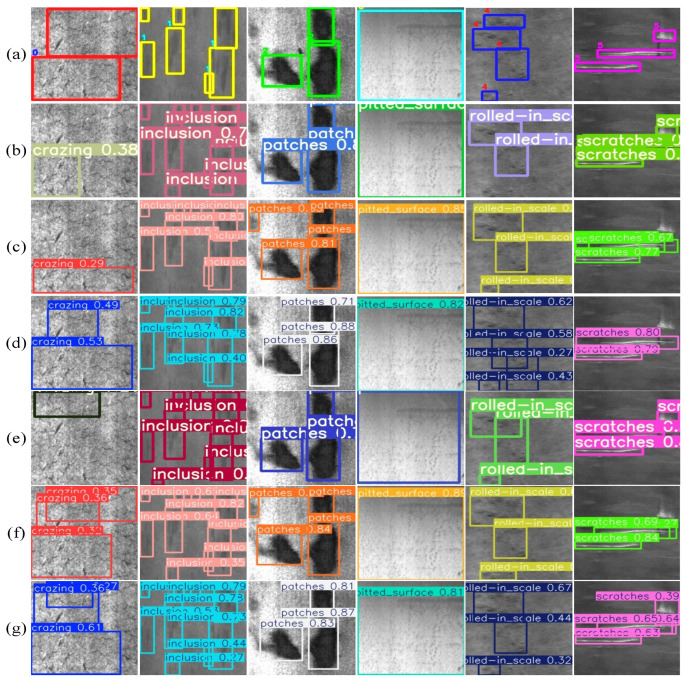
The prediction results for defects. (**a**) Ground truth. (**b**) YOLOv5s. (**c**) YOLOv8n. (**d**) YOLOv11n. (**e**) RDD-YOLO. (**f**) WSS-YOLO. (**g**) LCED-YOLO.

To validate the detection ability of the LCED-YOLO, we conducted a visual experiment of the detection effect. In the experiment, the LCED-YOLO model was compared with advanced detection models such as YOLOv5s, YOLOv8n, YOLOv11n, RDD-YOLO, and WSS-YOLO. As shown in Figure 9, in the experiment, a total of 6 different types of defects were selected; the prediction results were compared with ground truth boxes. Compared with other methods, LCED-YOLO shows excellent detection ability and can complete the detection task efficiently and accurately.

#### 4.4.2. Comparisons with Other Methods on GC10-DET

To further verify the effectiveness and generalization ability of LCED-YOLO, the model parameters are kept unchanged, and the verification is performed on the GC10-DET dataset. The specific results are shown in Table 3 and Table 4.

Compared with other advanced methods, LCED-YOLO showed the best performance, reaching 70.3% mAP50. SSD, Faster R-CNN, RT-DETR, and YOLOv5s show relatively low performance in the GC10-DET dataset. Models such as YOLOv8n, YOLOv11n, RDD-YOLO, and WSS-YOLO achieve higher mAP50, but their model sizes are relatively large and are not suitable for application in actual industrial environments. LCED-YOLO has obvious advantages, such as higher detection accuracy and lower model parameters and computational complexity. These results validate the model’s enhanced performance in practical industrial defect detection applications, establishing a robust basis for quality assurance in manufacturing workflows.

### 4.5. Ablation Experiments

This section performs ablation experiments on the proposed enhanced approach using the NEU-DET dataset to validate its efficacy. Furthermore, experimental comparisons were made regarding the combination of multi-scale pooling kernels within the C3K2-MSE module and the choice of the model’s loss function. For clarity in presenting the ablation results, seven experimental ablation schemes are abbreviated as follows:

The Baseline using the C3K2-MSE module is called E-YOLO.

The Baseline using the LDConv module is called D-YOLO.

The Baseline using the lightweight detection head is called L-YOLO.

The Baseline using Focaler-CIoU is called C-YOLO.

The Baseline using C3K2-MSE and LDConv modules is called ED-YOLO.

The Baseline using C3K2-MSE, LDConv, and lightweight detection head modules is called LED-YOLO.

The Baseline using C3K2-MSE, LDConv, a lightweight detection head, and Focaler-CIoU is called LCED-YOLO.

According to Table 5, the experimental results confirm the effectiveness of the proposed method. The LCED-YOLO model improves mAP50 by 2.6% compared to the Baseline. E-YOLO uses the C3K2-MSE module, and the model mAP50 is increased by 2.1%, which highlights the effectiveness of C3K2-MSE in capturing edge details of defects. D-YOLO introduces the LDConv module. While there is a marginal decline in detection accuracy, the model achieves a reduction of roughly 7.7% in parameters and 3.1% in computational complexity. By utilizing a lightweight decoupled head, L-YOLO improves mAP50 from 77.2% to 79%, reduces parameters by 15.3%, and reduces FLOPS to 5.2 G. This paper introduces a lightweight decoupled head that enhances model accuracy while simultaneously decreasing both parameters and computational complexity. Through the application of Focaler-CIoU for regression loss in bounding box prediction, C-YOLO improves its mAP50 by 1.1% while maintaining the same model parameters and computational load. This result indicates that Focaler-CIoU effectively strengthens the model’s ability to process imbalanced data samples. ED-YOLO uses both C3K2-MSE and LDConv modules to achieve 77.3% mAP50, which optimizes the model’s parameters. LDConv reduces the learning of positional offsets at each location via linearization, substituting downsampling techniques in the neck network to reduce the model’s parameters and computational complexity. It exhibits a robust capability for targets with significant feature variations, offering flexibility in response to deformations and local changes. Nevertheless, for targets that are less sensitive to feature variations, LDConv’s capacity for capturing global feature information is comparatively limited, potentially compromising the model’s detection performance. LED-YOLO uses C3K2-MSE, LDConv, and a lightweight decoupling head to achieve 78.2% mAP50 and 158 FPS, while optimizing the parameters and computational complexity of the model. Finally, the proposed method is integrated into LCED-YOLO, with mAP50 of 79.8%. The total number of parameters in the model is 2.1 M, representing a 19.2% reduction from the baseline. The computational complexity (FLOPS) is 5.0 G, a 23.1% decrease from the baseline, and the model achieves an inference speed of 151 FPS. In summary, this experiment proves the excellent detection performance of the LCED-YOLO model.

In the MSE module, it is necessary to conduct experiments to determine the optimal size for combining multi-scale pooling kernels. To ensure a sufficient and reasonable number of feature channels from the output of each convolutional layer, we experimented with the three combination sizes [3], [3, 6], and [3, 6, 9, 12]. The combination with the most significant impact on model performance was selected, and the results are presented in Table 6.

As shown in Table 6, the model performs optimally with the size combination [3, 6, 9, 12]. Conversely, the combinations [3] and [3, 6] result in a diminished capacity for capturing global feature information due to a higher number of channels, thereby compromising the model’s detection performance. A larger channel count also leads to an increase in the number of model parameters. Consequently, the [3, 6, 9, 12] combination is selected for use in the MSE module.

In the model, different loss functions will affect its performance. Therefore, when selecting a loss function, experiments are required to determine the best choice. This paper compares Focal-CIoU with other mainstream loss functions while keeping other conditions constant. From the results in Table 7, it can be seen that Focal-CIoU’s mAP50 is 79.8%, which is 1.7%, 1.0%, 0.5%, 0.7%, 1.2%, and 0.2% higher than the other loss functions, respectively. The introduction of Focal-CIoU enables the model to achieve the best overall performance. This indicates that compared to other loss functions, Focal-CIoU is the most suitable loss function for this model.

## 5. Conclusions

This paper introduces the LCED-YOLO model for detecting defects on steel surfaces. Firstly, the C3K2-MSE module is designed to enhance the model’s capacity to extract edge information, which in turn enhances the model’s detection accuracy for complex objects. Secondly, LDConv is introduced to lightweight the neck structure of the model, which effectively reduces the model’s parameter and computational complexity. Thirdly, a lightweight decoupling head is designed. Through the grouping and refinement of feature information, the model’s detection performance is significantly improved while achieving a lightweight architecture. Finally, the CIoU loss is optimized through the introduction of learnable attention factors, improving the model’s adaptability to imbalanced samples and thereby boosting its overall performance. LCED-YOLO on NEU-DET is 79.8% mAP50, Params is 2.1 M, FLOPS is 5.0 G, FPS is 151; on GC10-DET, the precision reaches 70.3% mAP50 and the FPS is 188. Compared with other excellent models, it demonstrated superior overall performance. In summary, the model demonstrates strong performance in detection accuracy, parameters, and computational complexity, though opportunities for enhancement remain. The next research focus should be on maintaining the model detection ability while optimizing the network structure to achieve a more lightweight effect and make it easier to apply in actual industrial settings.

## Figures and Tables

**Table 1:** Results on the NEU-DET dataset.

Method	AP50%						mAP50%
	Crs	In	Pa	Ps	Rs	Sc	
SSD	41.5%	72.9%	92.7%	89.0%	61.6%	78.2%	72.7%
Faster R-CNN	39.2%	73.1%	94.2%	88.4%	57.6%	97.3%	74.9%
RT-DETR	40.7%	75.4%	92.5%	76.1%	69.3%	89.8%	74.0%
YOLOV5s	39.5%	77.6%	92.9%	88.0%	58.9%	95.1%	75.3%
YOLOV8n	42.9%	74.0%	94.2%	84.8%	73.8%	92.4%	77.0%
YOLOV11n	43.8%	78.3%	94.5%	84.9%	67.1%	94.7%	77.2%
RDD-YOLO	50.8%	78.3%	93.5%	85.4%	63.6%	90.5%	77.0%
WSS-YOLO	51.4%	79.1%	93.9%	86.8%	74.5%	93.7%	79.9%
LCED-YOLO	48.2%	82.6%	94.5%	89.1%	70.6%	93.5%	79.8%

**Table 2:** Comparison of results on the NEU-DET dataset.

Method	Precision	Recall	mAP50%	mAP (50–95)%	Params/M	FLOPS/G	FPS
SSD	76.6%	60.2%	72.7%	33.8%	50.2	360.9	106
Faster R-CNN	54.9%	89.9%	74.9%	37.5%	136.7	369.7	32
RT-DETR	78.5%	65.4%	74.0%	43.3%	19.8	57.0	144
YOLOV5s	67.2%	75.9%	75.3%	39.2%	7.1	16.5	153
YOLOV8n	70.9%	72.2%	77.0%	43.0%	3.2	8.2	144
YOLOV11n	74.8%	72.4%	77.2%	45.8%	2.6	6.5	156
RDD-YOLO	72.2%	73.0%	77.0%	43.3%	74.2	190.0	57
WSS-YOLO	77.6%	71.2%	79.9%	46.9%	4.5	9.6	69
LCED-YOLO	69.7%	76.8%	79.8%	46.8%	2.1	5.0	151

**Table 3:** Results on the GC10-DET dataset.

Method	AP50%										mAP50%
	Pu	Wl	Cg	Ws	Os	Ss	In	Rp	Crs	Wf	
SSD	16.4%	46.8%	82.7%	56.6%	21.0%	12.2%	7.4%	24.6%	39.7%	95.8%	40.3%
Faster R-CNN	62.5%	44.1%	94.4%	70.8%	38.3%	37.0%	18.2%	36.3%	37.3%	48.1%	48.7%
RT-DETR	84.3%	98.5%	94.6%	72.7%	56.3%	52.9%	39.1%	33.8%	44.1%	85.2%	66.1%
YOLOV5s	89.0%	84.1%	96.0%	66.2%	57.4%	51.2%	40.1%	37.6%	50.0%	77.4%	64.9%
YOLOV8n	99.5%	95.5%	95.9%	68.8%	64.5%	61.2%	25.0%	17.4%	75.0%	89.1%	69.2%
YOLOV11n	99.2%	90.8%	97.6%	73.4%	66.9%	62.9%	26.5%	14.3%	46.9%	91.1%	67.0%
RDD-YOLO	91.4%	84.0%	96.8%	74.6%	57.4%	57.7%	40.1%	69.4%	23.3%	80.9%	67.6%
WSS-YOLO	99.2%	93.6%	97.2%	73.6%	68.5%	62.1%	23.3%	14.4%	68.9%	93.6%	69.4%
LCED-YOLO	99.3%	96.6%	94.9%	73.8%	70.2%	68.6%	32.5%	15.8%	59.0%	92.6%	70.3%

**Table 4:** Comparison of results on the DC10-DET dataset.

Method	Precision	Recall	mAP50%	mAP (50–95)%	Params/M	FLOPS/G	FPS
SSD	73.4%	29.2%	40.3%	29.2%	50.2	360.9	138
Faster R-CNN	47.6%	59.1%	48.7%	18.4%	136.7	369.7	47
RT-DETR	73.9%	64.9%	66.1%	32.1%	19.8	57.0	176
YOLOV5s	70.2%	60.5%	64.9%	29.9%	7.1	16.5	244
YOLOV8n	66.4%	67.5%	69.2%	34.8%	3.2	8.2	238
YOLOV11n	65.6%	63.5%	67.0%	34.0%	2.6	6.5	227
RDD-YOLO	65.5%	68.7%	67.6%	30.4%	74.2	190.0	102
WSS-YOLO	73.7%	65.1%	69.4%	35.9%	4.5	9.6	166
LCED-YOLO	74.2%	63.6%	70.3%	35.7%	2.1	5.0	188

**Table 5:** Ablation experiment results.

Method	Precision	Recall	mAP50%	mAP (50–95)%	Params/M	FLOPS/G	FPS
Baseline	74.8%	72.4%	77.2%	45.8%	2.6	6.5	156
E-YOLO	75.7%	73.2%	79.3%	47.0%	2.6	6.4	151
D-YOLO	77.4%	65.7%	76.9%	45.1%	2.4	6.2	149
L-YOLO	74.1%	75.3%	79.0%	45.8%	2.2	5.2	153
C-YOLO	72.9%	75.3%	78.3%	45.4%	2.6	6.5	166
ED-YOLO	69.5%	72.7%	77.3%	42.5%	2.4	6.2	140
LED-YOLO	74.2%	73.0%	78.2%	45.4%	2.1	5.0	158
LCED-YOLO	69.7%	76.8%	79.8%	46.8%	2.1	5.0	151

**Table 6:** Comparison of multi-scale pooling kernel combinations in C3K2-MSE.

Pooling Kernel Size	Precision	Recall	mAP50%	mAP (50–95)%	Params/M
[3]	78.2%	70.4%	77.9%	42.3%	2.532
[3, 6]	69.3%	77.9%	78.6%	43.5%	2.532
[3, 6, 9, 12]	75.7%	73.2%	79.3%	47.0%	2.531

**Table 7:** Comparison of different loss functions.

Metrics	Precision	Recall	mAP50%	mAP (50–95)%
Focaler-DIoU	73.8%	75.7%	78.1%	43.1%
Focaler-EIoU	75.7%	74.7%	78.8%	48.0%
Focaler-GIoU	75.1%	74.6%	79.3%	47.6%
Focaler-SIoU	73.9%	76.3%	79.1%	43.8%
Focaler-ShapeIoU	76.9%	71.1%	78.6%	47.7%
MPDIoU	71.9%	77.3%	79.6%	42.6%
Focaler-CIoU	69.7%	76.8%	79.8%	46.8%

## Data Availability

The data that support the findings of this study are available from the corresponding author upon reasonable request.
